# Supplementary material for: Limited structural reliability in public educational prediction benchmarks: a four-dimension audit of seven datasets
Source: Sci Rep. 2026 Sep 5;16:28593. doi: 10.1038/s41598-026-69629-6 (PMC13574758; doi:10.1038/s41598-026-69629-6)
Supplement: Supplementary file 1 [file 41598_2026_69629_MOESM1_ESM.pdf]

Supplementary Materials: A Four-Dimension Pre-Modeling Audit Protocol for Educational Prediction Benchmarks

Yan Ma[1,2] Lizhuo Zhang[3,*]

[1] School of Foreign Studies, Changsha University of Science and Technology, Changsha 410114, China

[2] School of Education, Hunan Agricultural University, Changsha 410128, China

[3] School of Information and Intelligence, Hunan Agricultural University, Changsha 410128, China

[*] Correspondence: zhanglizhuo@hunau.edu.cn

This document contains supplementary tables and figures for the main manuscript.

S1 Model-Complexity Ablation

Table S1: Model-complexity ablation: instability ratio (I) and beat rate across linear and nonlinear models on 100 repeated 80/20 splits.

Dataset	Model	MAE (mean ± SD)	R^2 (mean)	r (mean)	I	Beat rate
MM-TBA	Linear	0.795 ± 0.089	-0.067	0.178	2.21	0.80
	Ridge	0.793 ± 0.089	-0.061	0.183	2.09	0.81
	Random Forest	0.815 ± 0.081	-0.063	0.196	3.96	0.65
	Gradient Boosting	0.856 ± 0.095	-0.227	0.129	4.60	0.45
Higher Ed	Linear	1.695 ± 0.199	0.041	0.469	0.90	0.83
	Ridge	1.686 ± 0.195	0.054	0.470	0.84	0.83
	Random Forest	1.193 ± 0.130	0.521	0.744	0.18	1.00
	Gradient Boosting	1.273 ± 0.146	0.464	0.711	0.23	1.00
xAPI-Edu	Linear	0.363 ± 0.025	0.614	0.786	0.12	1.00
	Ridge	0.357 ± 0.023	0.626	0.799	0.11	1.00
	Random Forest	0.308 ± 0.023	0.682	0.830	0.09	1.00
	Gradient Boosting	0.327 ± 0.024	0.663	0.818	0.10	1.00
Entrance Exam	Linear	0.598 ± 0.033	0.438	0.670	0.12	1.00
	Ridge	0.594 ± 0.032	0.448	0.676	0.11	1.00
	Random Forest	0.612 ± 0.036	0.372	0.628	0.14	1.00
	Gradient Boosting	0.595 ± 0.033	0.442	0.671	0.12	1.00
UCI Student	Linear	2.011 ± 0.148	0.242	0.530	0.36	1.00
	Ridge	2.010 ± 0.148	0.263	0.530	0.36	1.00
	Random Forest	2.005 ± 0.151	0.285	0.548	0.37	1.00
	Gradient Boosting	2.046 ± 0.151	0.257	0.531	0.41	1.00
Dropout	Linear	0.206 ± 0.006	0.651	0.807	0.021	1.00
	Ridge	0.206 ± 0.006	0.651	0.807	0.021	1.00
	Random Forest	0.153 ± 0.006	0.684	0.828	0.019	1.00
	Gradient Boosting	0.160 ± 0.006	0.692	0.832	0.019	1.00
OULAD	Linear	0.296 ± 0.002	0.471	0.687	0.010	1.00
	Ridge	0.296 ± 0.002	0.471	0.687	0.010	1.00
	Random Forest	0.193 ± 0.003	0.577	0.761	0.009	1.00
	Gradient Boosting	0.203 ± 0.002	0.610	0.781	0.008	1.00

S2 EDA Summary

Table S2: Exploratory data analysis summary for the seven audited datasets. All releases contain zero missing values. The datasets that fail the audit (MM-TBA, Entrance Exam, Higher Ed, UCI Student) each fail at least one dimension, yet none show distinctive EDA red flags—such as extreme imbalance or redundant features—that would pre-emptively disqualify them; the fragility is only revealed through the four-dimension audit.

Dataset	N	Feat.	Target	Dist.	n_{grp}	Grp sz (min/mean/max)
OULAD	32,593	44	Pass=1, Fail/W=0	0.47:0.53	22	365/1482/2498
Dropout	3,630	36	Grad=1, Dropout=0	0.61:0.39	17	9/214/666
Entrance Exam	666	49	4-class ordinal	24%:32%:30%:15%	3	21/222/396
UCI Student	649	56	Grade $G3$ [0,20]	$\mu = 11.9$, $\sigma = 3.2$	2	226/324/423
xAPI-Edu	480	72	3-class ordinal	26%:44%:30%	12	19/40/95
MM-TBA	186	13	Lecture quality [0.5,7.5]	$\mu = 6.0$, $\sigma = 1.0$	0	—
Higher Ed	145	31	Grade [0,7]	$\mu = 3.5$, $\sigma = 2.2$	9	2/16/66

S3 Train-Test R^2 Gap

Table S3: Train-test R^2 gap across 100 repeated 80/20 splits. Within each split, Z-score parameters are fitted exclusively on the training partition and applied to the test partition. The gap (train R^2 – test R^2) quantifies how much of the apparent iid signal is lost on held-out samples from the same distribution. On UCI Student, the linear-model train-test gap (0.105) is small relative to the iid-to-group-holdout collapse (0.242 to -0.097), confirming that the group-holdout failure is not attributable to ordinary overfitting. On OULAD, both gaps remain small.

Dataset	Model	Train R^2 mean	Test R^2 mean	Gap mean	Gap SD
UCI Student	Linear	0.347	0.242	0.105	0.091
	Ridge	0.347	0.242	0.105	0.091
	RF	0.895	0.265	0.630	0.084
	GBT	0.603	0.259	0.345	0.090
Higher Ed	Linear	0.460	-0.092	0.552	0.335
	Ridge	0.459	-0.079	0.539	0.328
	RF	0.877	0.085	0.793	0.143
	GBT	0.850	0.054	0.796	0.171
OULAD	Linear	0.471	0.464	0.008	0.026
	Ridge	0.471	0.464	0.008	0.026
	RF	0.933	0.573	0.360	0.024
	GBT	0.629	0.587	0.042	0.026

S4 Group-Holdout Uncertainty

Table S4: Across-group variation in group-holdout R^2. The SD quantifies how consistent the group-holdout performance is across held-out groups. For UCI Student (2 schools), the linear-model SD of 0.109 with a narrow range (−0.206 to 0.012) confirms uniform collapse rather than group-specific failure. For OULAD (22 cohorts), the across-cohort SD of 0.162 and full range (0.041 to 0.654) reflect positive—though variable—cross-group generalization.

Dataset	Model	Groups tested	Mean R^2	SD R^2	Min / Max R^2
UCI Student	Linear	2	−0.097	0.109	−0.206 / 0.012
	Ridge	2	−0.094	0.106	−0.201 / 0.012
	RF	2	0.018	0.109	−0.091 / 0.127
	GBT	2	−0.057	0.136	−0.193 / 0.079
Higher Ed	Linear	4	−8.79	6.39	−18.65 / −0.975
	Ridge	4	−8.58	6.27	−18.35 / −0.969
	RF	4	−13.0	14.0	−35.59 / −0.169
	GBT	4	−10.6	11.3	−29.11 / −0.597
OULAD	Linear	22	0.410	0.162	0.041 / 0.654
	Ridge	22	0.410	0.162	0.041 / 0.654
	RF	22	0.482	0.211	−0.052 / 0.747
	GBT	22	0.533	0.165	0.039 / 0.752

S5 Linear-Model Coefficient Stability

Table S5: Top-10 linear-model standardized coefficient stability across 100 repeated 80/20 splits for the three illustrative datasets. The sign-flip rate is the proportion of splits where the coefficient sign differs from the sign of the mean coefficient. On OULAD, all top-10 features show zero sign flips with low SD, indicating stable directional relationships. On UCI Student, sign-flip rates are near zero for most top features but f9 shows a 3% rate and f2 a 4% rate, indicating occasional directional instability; SDs are proportionally larger than on OULAD. On Higher Ed, sign-flip rates are near zero for most top features (f9: 2%) with SDs reflecting greater uncertainty about effect magnitude.

Dataset	Feature	Mean coef	SD coef	Sign-flip rate
UCI Student	f5	−0.862	0.063	0.00
	f4	0.393	0.057	0.00
	f49	0.270	0.032	0.00
	f48	−0.270	0.032	0.00
	f11	−0.253	0.051	0.00
	f30	0.229	0.061	0.00
	f33	−0.217	0.054	0.00
	f9	−0.202	0.097	0.03
	f2	0.199	0.086	0.04
	f22	0.190	0.047	0.00
Higher Ed	f28	0.669	0.123	0.00
	f0	−0.662	0.134	0.00
	f17	0.618	0.121	0.00
	f1	0.506	0.119	0.00
	f8	−0.418	0.103	0.00
	f25	0.402	0.119	0.00
	f4	0.401	0.132	0.00
	f2	0.332	0.108	0.00
	f15	−0.304	0.103	0.00
	f9	0.300	0.109	0.02
OULAD	f3	0.175	0.005	0.00
	f2	0.162	0.006	0.00
	f4	0.114	0.003	0.00
	f1	−0.080	0.005	0.00
	f0	−0.039	0.003	0.00
	f5	−0.024	0.003	0.00
	f10	0.022	0.001	0.00
	f11	−0.022	0.001	0.00
	f29	−0.015	0.002	0.00
	f27	0.013	0.002	0.00

S6 Random Forest Importance Stability

Table S6: Top-10 Random Forest feature importance stability across 100 repeated 80/20 splits. The coefficient of variation (CV = SD / mean) quantifies relative uncertainty. On OULAD, all top-10 CVs are below 0.12, indicating stable importance rankings. On UCI Student, CVs range from 0.08 to 0.20; on Higher Ed, from 0.18 to 0.41, reflecting greater split sensitivity in smaller datasets.

Dataset	Feature	Mean importance	SD importance	CV
UCI Student	f5	0.193	0.015	0.076
	f12	0.068	0.007	0.100
	f8	0.047	0.006	0.125
	f0	0.044	0.005	0.107
	f7	0.039	0.005	0.121
	f4	0.038	0.006	0.145
	f2	0.036	0.004	0.123
	f10	0.036	0.005	0.130
	f9	0.036	0.007	0.198
	f6	0.034	0.005	0.155
Higher Ed	f28	0.131	0.028	0.212
	f1	0.073	0.030	0.409
	f3	0.064	0.016	0.243
	f27	0.053	0.014	0.255
	f10	0.046	0.010	0.211
	f12	0.044	0.008	0.178
	f11	0.040	0.009	0.212
	f16	0.039	0.008	0.205
	f0	0.038	0.010	0.255
	f7	0.037	0.011	0.291
OULAD	f3	0.512	0.006	0.012
	f4	0.137	0.004	0.029
	f2	0.071	0.003	0.044
	f5	0.064	0.002	0.029
	f1	0.059	0.002	0.037
	f0	0.012	0.001	0.073
	f29	0.007	0.001	0.082
	f27	0.006	0.001	0.080
	f35	0.006	0.001	0.093
	f36	0.005	0.001	0.096

S7 Feature-Name Mapping

Table S7: Mapping from feature codes used in Supplementary Tables S5–S6 to human-readable feature names for the three datasets included in the feature-attribution stability analysis. Feature codes correspond to zero-indexed column positions in the preprocessed feature matrix after one-hot encoding. Group-identifier columns (school for UCI Student, Course ID for Higher Ed) are excluded from the feature matrix, so their corresponding features do not appear in the tables.

Dataset	Code	Feature name
UCI Student	f0	age
	f2	Fedu
	f4	studytime
	f5	failures
	f6	famrel
	f7	freetime
	f8	goout
	f9	Dalc
	f10	Walc
	f11	health
	f12	absences
	f22	Mjob_health
	f30	Fjob_teacher
	f33	reason_other
	f48	higher_no
	f49	higher_yes
Higher Ed	f0	Student Age
	f1	Sex
	f2	Graduated high-school type
	f3	Scholarship type
	f4	Additional work
	f7	Total salary if available
	f8	Transportation to the university
	f9	Accomodation type in Cyprus
	f10	Mother's education
	f11	Father's education
	f12	Number of sisters/brothers (if available)
	f15	Father's occupation
	f16	Weekly study hours
	f17	Reading frequency (non-scientific books/journals)
	f25	Listening in classes
	f27	Flip-classroom
	f28	Cumulative grade point average in the last semester (/4.00)
OULAD	f0	num_of_prev_attempts
	f1	vle_total_clicks
	f2	vle_active_weeks
	f3	assessment_count
	f4	assessment_score_mean
	f5	assessment_score_std
	f10	gender_F
	f11	gender_M
	f27	highest_education_A Level or Equivalent
	f29	highest_education_Lower Than A Level
	f35	imd_band_20–30%
	f36	imd_band_30–40%

S8 Cross-Model Attribution Agreement

Table S8: Cross-model Spearman rank correlation between linear-model absolute standardized coefficients and Random Forest impurity-based importances across 100 repeated 80/20 splits. ρ_{all} uses all features; ρ_{top10} uses the top-10 features by linear absolute coefficient in each split. On OULAD, cross-model agreement is high and stable across splits ($\rho_{\text{top10}} = 0.817$, SD 0.065). On UCI Student and Higher Ed, agreement is weaker and more variable, consistent with their lower-readiness profiles.

Dataset	p	ρ_{all}		ρ_{top10}	
		Mean	SD	Mean	SD
UCI Student	54	0.496	0.093	0.516	0.242
Higher Ed	30	0.285	0.126	0.498	0.245
OULAD	44	0.752	0.048	0.817	0.065

S9 Supplementary Figures

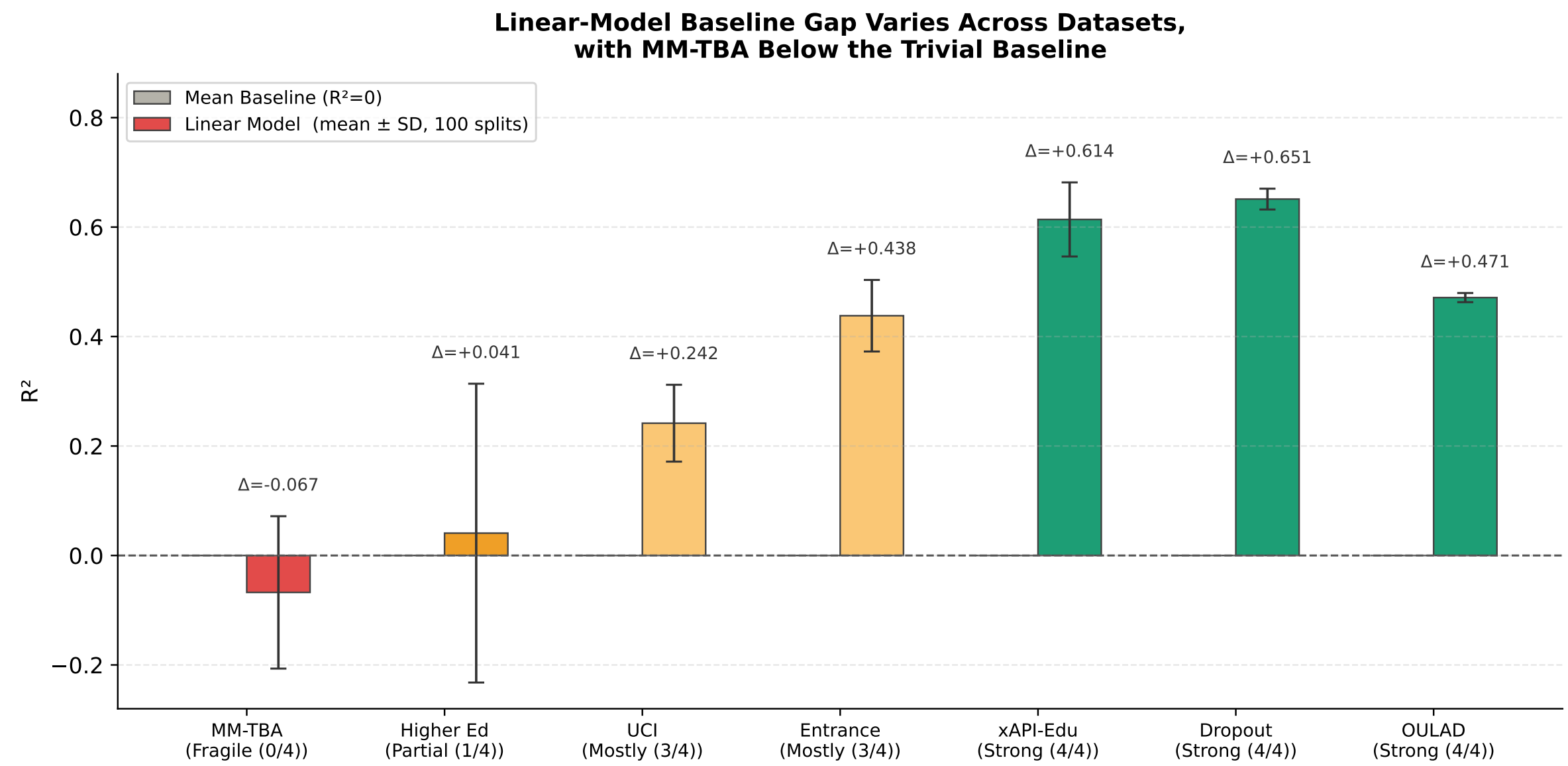

Fig S1: Baseline gap: linear-model mean R^2 (with cross-split SD error bars) over 100 random 80/20 splits for the seven datasets, ordered by readiness profile. The grey reference bar denotes the trivial mean baseline ($R^2 = 0$). The figure complements Table 2 by showing the dispersion of split-level R^2 around the reported mean; MM-TBA and Higher Ed have means near or below zero with relatively wide spread, consistent with their Fragile / Partial classifications.

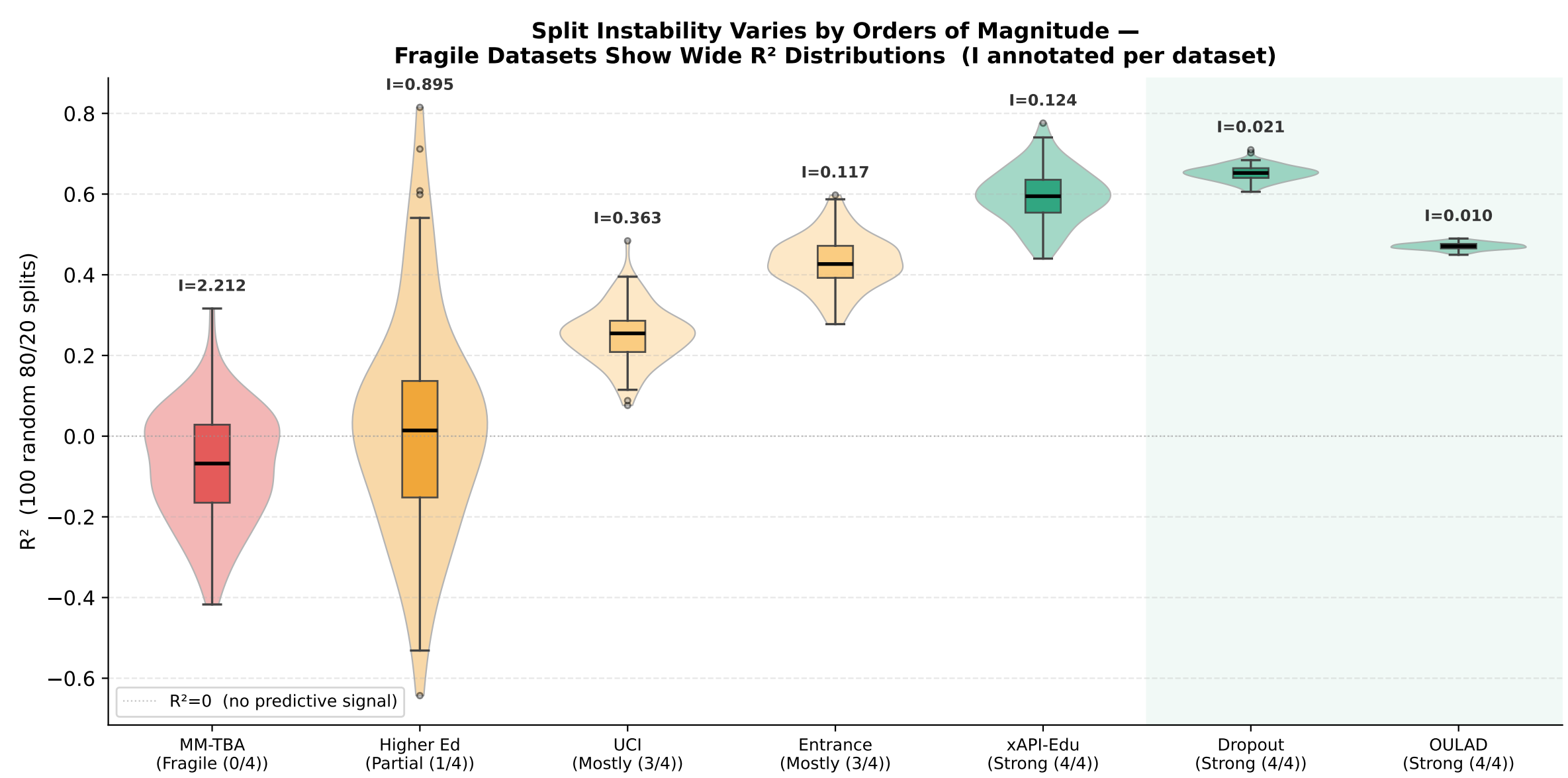

Fig S2: Split instability: violin and box plots of the linear-model R^2 across 100 random 80/20 splits for each dataset, ordered by readiness profile. The instability ratio I is annotated above each violin. Strong datasets (OULAD, Dropout) have tight distributions ($I < 0.03$); MM-TBA's distribution is broad and centred on negative R^2 ($I = 2.21$), reproducing Table 2 visually.

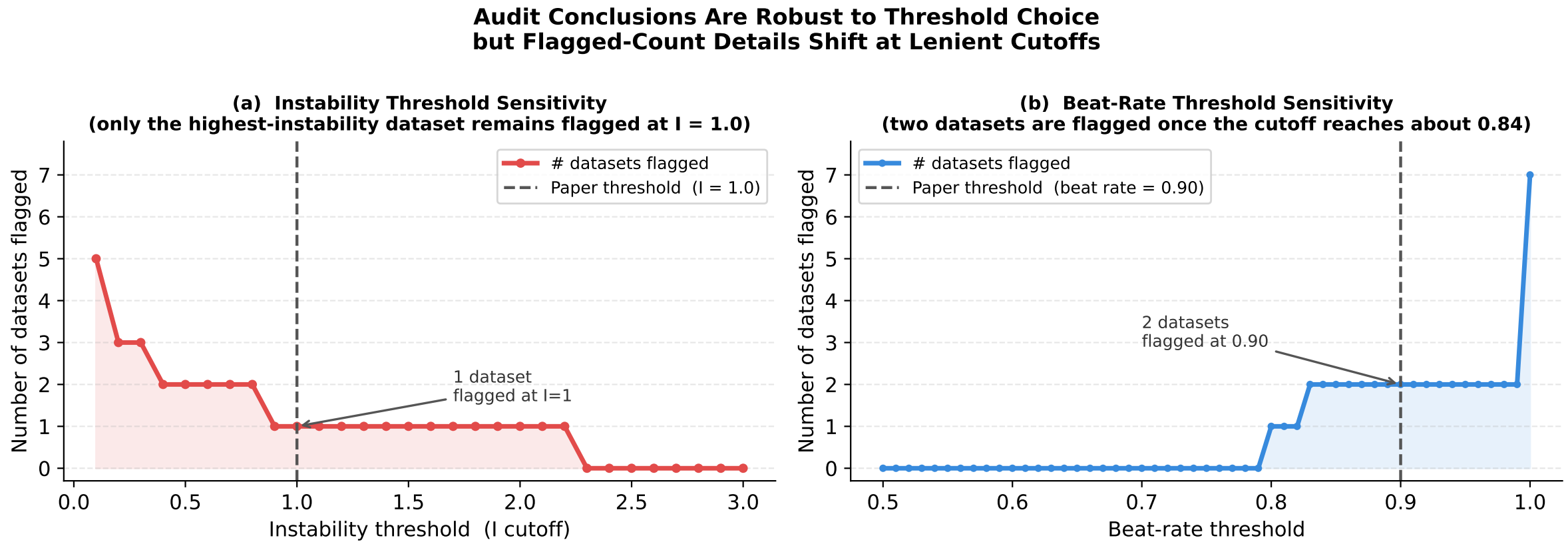

Fig S3: Threshold-sensitivity sweeps for the two operational cutoffs used in the audit. Panel (a) varies the instability cutoff over $I \in [0.1, 3.0]$ and reports the number of datasets that would be flagged as failing split instability; the paper's choice of $I = 1.0$ flags one dataset (MM-TBA, with linear $I = 2.21$), while more stringent cutoffs below about 0.9 additionally flag Higher Ed. Panel (b) varies the beat-rate cutoff over $[0.5, 1.0]$; the paper's choice of 0.90 flags two datasets, whereas more lenient cutoffs below about 0.84 reduce the flagged count. The main qualitative pattern remains unchanged: MM-TBA is always the weakest case, and Higher Ed is the only borderline dataset near the operational thresholds.
